# Decellularized Pulmonary Homograft Repair of Idiopathic Main Pulmonary Artery Aneurysm in SC Hemoglobinopathy

**DOI:** 10.1016/j.jaccas.2026.106933

**Published:** 2026-02-13

**Authors:** Michael Hikaru Mikami, Clara Mayumi Mikami, Lucas do Valle Ciccozzi, Stephanie Senna da Silva, Paulo Roberto Slud Brofman, Claudinei Collatusso

**Affiliations:** a Cardiovascular Surgery, Hospital Santa Casa de Misericórdia de Curitiba, Curitiba, Brazil; b Faculdade Evangélica Mackenzie do Paraná, Curitiba, Brazil

**Keywords:** aneurysm, homograft, pulmonary artery, SC hemoglobinopathy

## Abstract

**Background:**

Pulmonary artery aneurysm is a rare and potentially fatal condition, often diagnosed incidentally. The lack of specific guidelines makes it difficult to define clear intervention criteria and the ideal reconstructive technique. Decellularized pulmonary homografts (DPHs) have emerged as a promising alternative, although they are still scarcely reported.

**Case Summary:**

A 48-year-old previously healthy man presented with mediastinal widening on a routine chest x-ray. Work-up revealed an idiopathic main pulmonary aneurysm measuring 52 mm, associated with moderate pulmonary valve stenosis. He underwent aneurysm resection and valve replacement with a DPH (size 25). The immediate postoperative course was satisfactory, but he developed severe pneumonia and was incidentally diagnosed with SC hemoglobinopathy.

**Discussion:**

The use of a DPH was appropriate in this setting, particularly considering the patient's age and the presence of SC hemoglobinopathy, a condition associated with increased risk of pulmonary hypertension, thrombotic events, and vascular inflammation. Follow-up demonstrated a competent graft with no thromboembolic complications, reinforcing the potential benefit of this technique.

**Take-Home Message:**

This case highlights the use of a DPH as a safe and effective surgical option for an idiopathic main pulmonary artery aneurysm, especially in patients with associated hematologic conditions, emphasizing the importance of strict follow-up and individualized therapeutic strategies.

## Visual Summary

**Figure:**
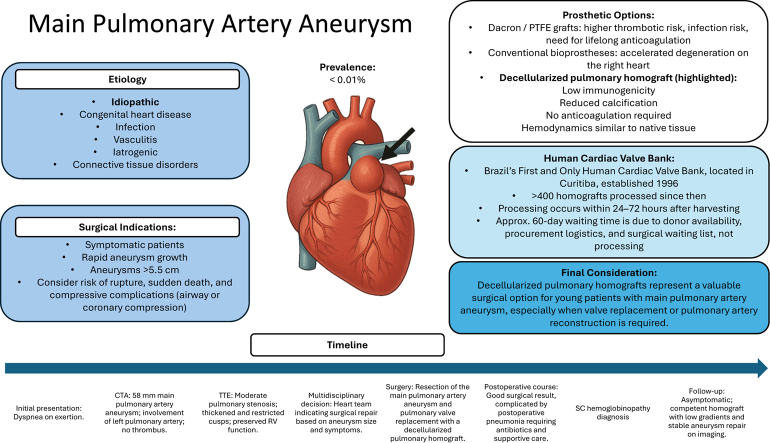

**Visual Summary:**
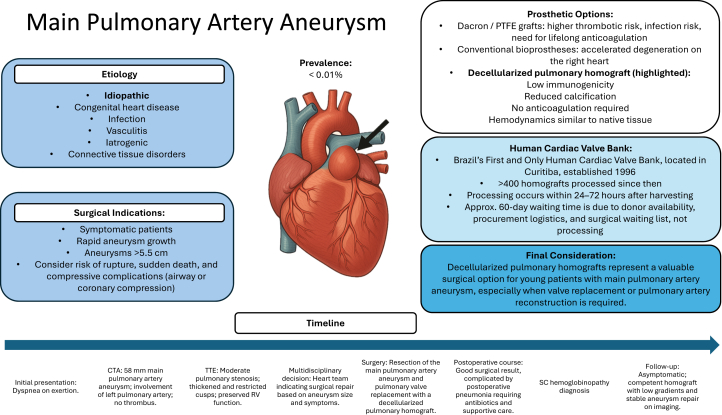
Timeline of Case Presentation CTA = computed tomography angiography; PTFE = polytetrafluoroethylene; RV = right ventricle; TTE = transthoracic echocardiography.

Pulmonary artery aneurysm is an uncommon entity, with a reported prevalence of <0.01%,^1^ and it carries the risk of serious complications such as rupture, dissection, or compression of adjacent mediastinal structures.^2^ Its etiology is multifactorial, including pulmonary hypertension, congenital heart disease, infections, vasculitis, and idiopathic causes.^3^ Given its rarity, there are no established guidelines for surgical indication or choice of reconstructive technique.^4^ Although pulmonary artery aneurysm or pseudoaneurysm has been reported in patients with beta-thalassemia major,^5^ to our knowledge, there are no reports of pulmonary artery aneurysm in patients with SC hemoglobinopathy. We present a case of idiopathic main pulmonary aneurysm treated with a decellularized pulmonary homograft (DPH) in a patient later diagnosed with SC hemoglobinopathy.

**Take-Home Messages**

• Idiopathic main pulmonary artery aneurysm is rare and challenging.
• Decellularized pulmonary homografts are a safe and effective option in young patients.
• Hematologic comorbidities require strict follow-up and individualized care.
• Multicenter studies are needed to establish surgical criteria and guide graft selection.

## History of Presentation

**Figure 1:**
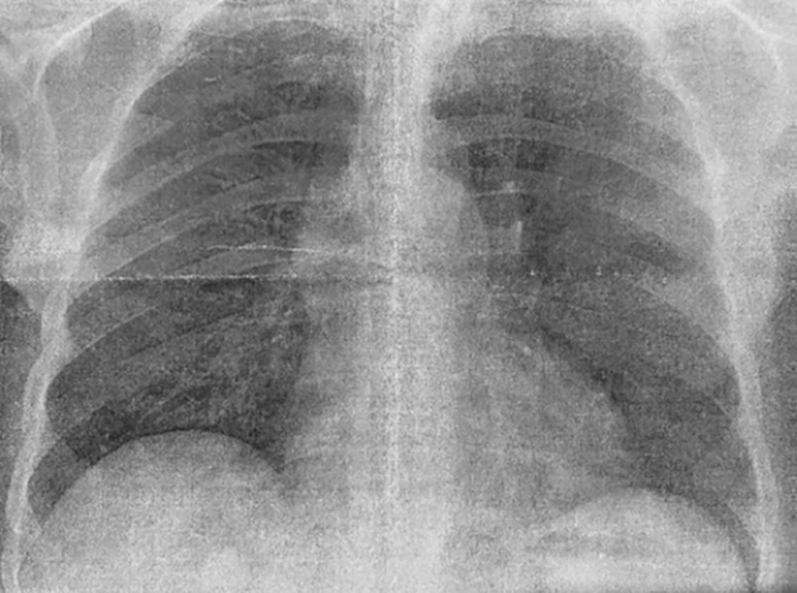
Chest X-Ray Demonstrating Mediastinal Widening in the Main Pulmonary Artery Region

A 48-year-old man with no previous comorbidities presented with mediastinal widening on occupational chest radiography (Figure 1). He reported atypical, nonanginal chest pain not related to exertion. Physical examination revealed good overall condition, with normal cardiopulmonary auscultation.

## Investigations

Electrocardiogram showed sinus rhythm with no significant abnormalities. Echocardiography revealed moderate pulmonary valve stenosis, with a maximum gradient of 45 mm Hg, thickened cusps with reduced opening, associated with right ventricular hypertrophy, and preserved right ventricular function. Left ventricular ejection fraction was 68%, with estimated main pulmonary artery (MPA) systolic pressure of 34 mm Hg (Table 1).

**Table 1:** Findings on Preoperative Transthoracic Echocardiogram Showing Moderate Pulmonary Valve Stenosis and Elevated Gradients

Measure	Value
Aortic root (mm)	32
Left atrium (mm)	42
Right ventricle (mm)	24
Interatrial septum (mm)	11
Posterior wall thickness (mm)	11
LV diastolic diameter (mm)	49
LV systolic diameter (mm)	30
Shortening fraction (%)	38
LVEF (Simpson%)	68
LV mass (g)/BSA	240
TAPSE (mm Hg)	—
PSAP (mm Hg)	34
Descriptions	
Tricuspid valve	Thickened leaflets, preserved, insufficient aperture.
Pulmonary valve	Thickened leaflets with decreased, competent aperture. Maximum gradient: 45 mm Hg, mean: 23 mm Hg, jet velocity: 3.3 mL/m^2^.
Conclusion	Left ventricle with cavity, preserved systolic function and contraction, altered relaxation. Enlarged atria. Mild mitral regurgitation. Mild tricuspid regurgitation. Hypertrophied right ventricle with preserved function. Moderate pulmonary stenosis.

BSA = body surface area; LV = left ventricle; LVEF = left ventricular ejection fraction; PSAP = pulmonary systolic artery pressure; TAPSE = tricuspid annular plane systolic excursion.

**Figure 2:**
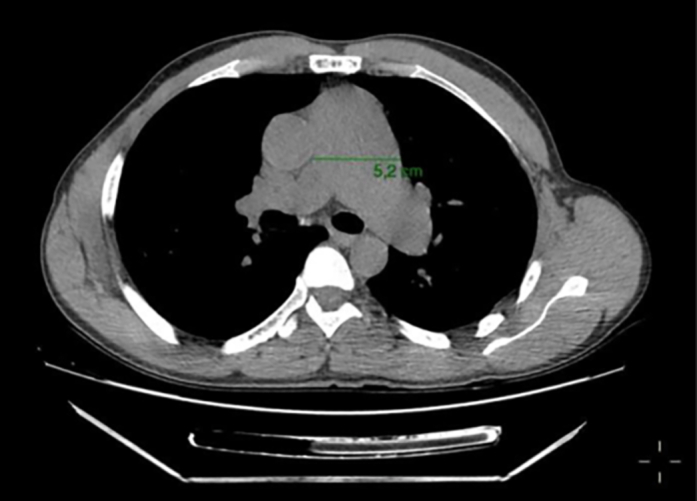
Chest Computed Tomography Scan Showing Main Pulmonary Artery Aneurysm With a 52-mm Diameter

**Figure 3:**
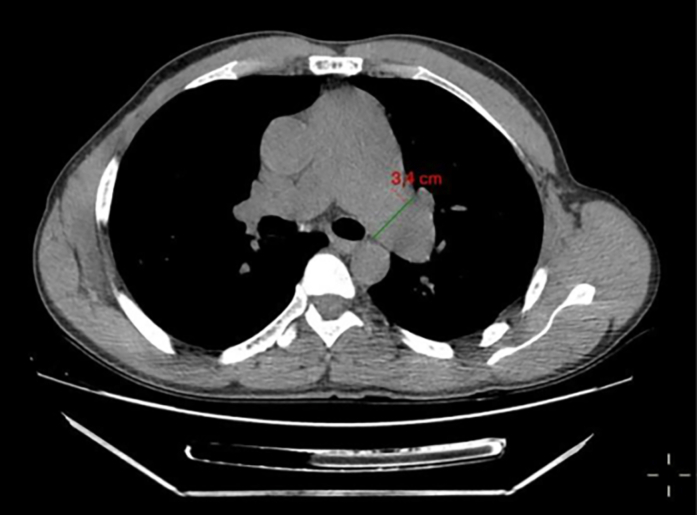
Chest Computed Tomography Scan Showing Left Pulmonary Artery Aneurysm With a 34-mm Diameter

**Figure 4:**
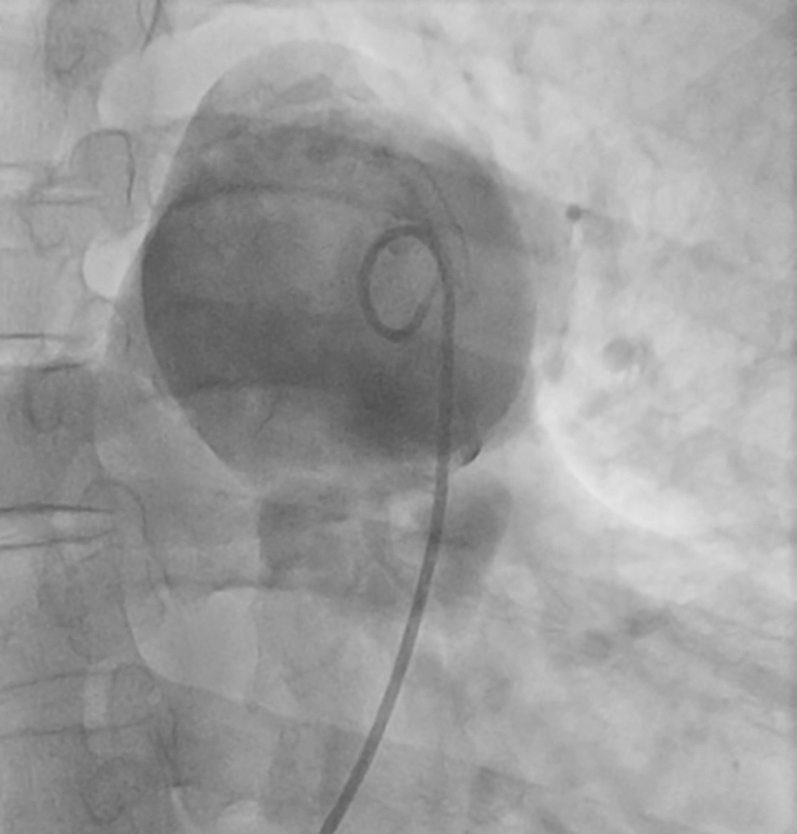
Aneurysmal Dilatation of the Main Pulmonary Artery Demonstrated During Cardiac Catheterization

Chest computed tomography angiography demonstrated a 52-mm MPA aneurysm (Figure 2), along with dilation of the left main branch (Figure 3). Cardiac catheterization confirmed a transvalvular gradient of 50 mm Hg, mean pulmonary capillary wedge pressure of 15 mm Hg, and no coronary artery disease (Figure 4).

## Differential Diagnosis

Considerations included the following:

• Idiopathic main pulmonary artery aneurysm
• Pulmonary hypertension–related dilation
• Congenital heart disease
• Vasculitis
• Infectious pseudoaneurysm
• Connective tissue disorders

## Management

**Figure 5:**
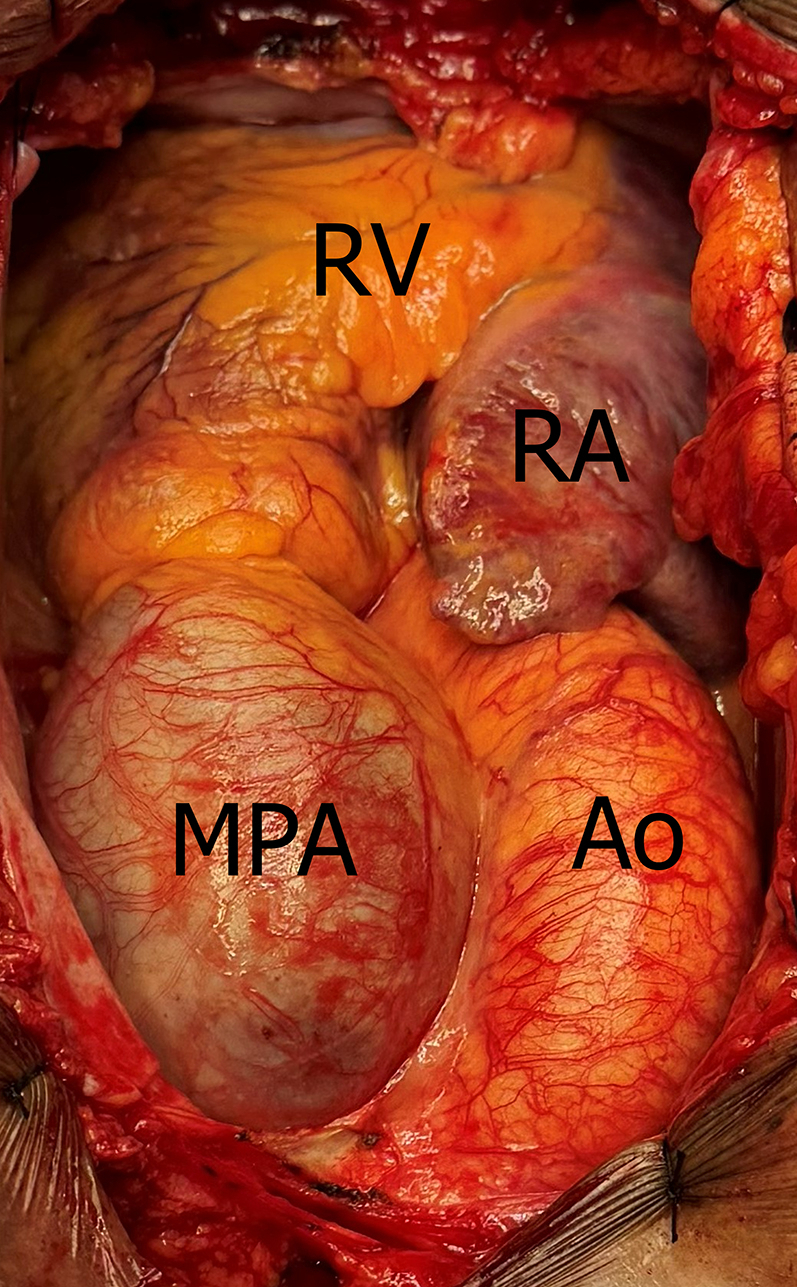
Aneurysmal Dilatation of the Main Pulmonary Artery on Surgical View Ao = aorta; MPA = main pulmonary artery; RA = right atrium; RV = right ventricle.

**Figure 6:**
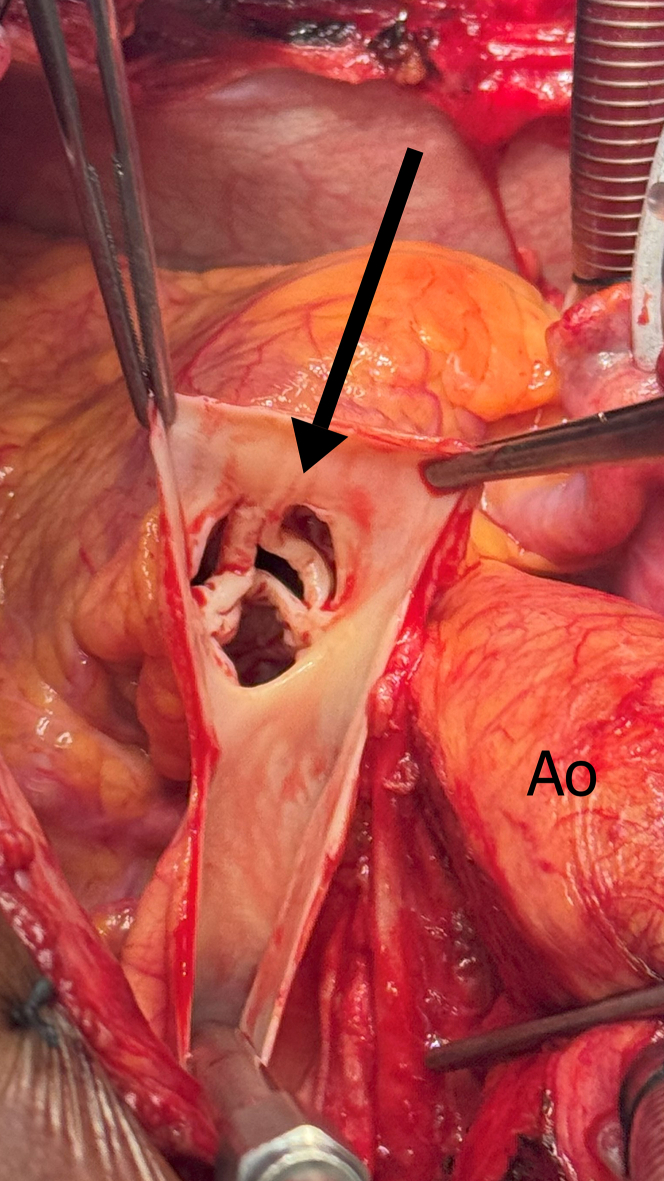
Pulmonary Valve Stenosis (Arrow) on Surgical View Ao = aorta.

**Figure 7:**
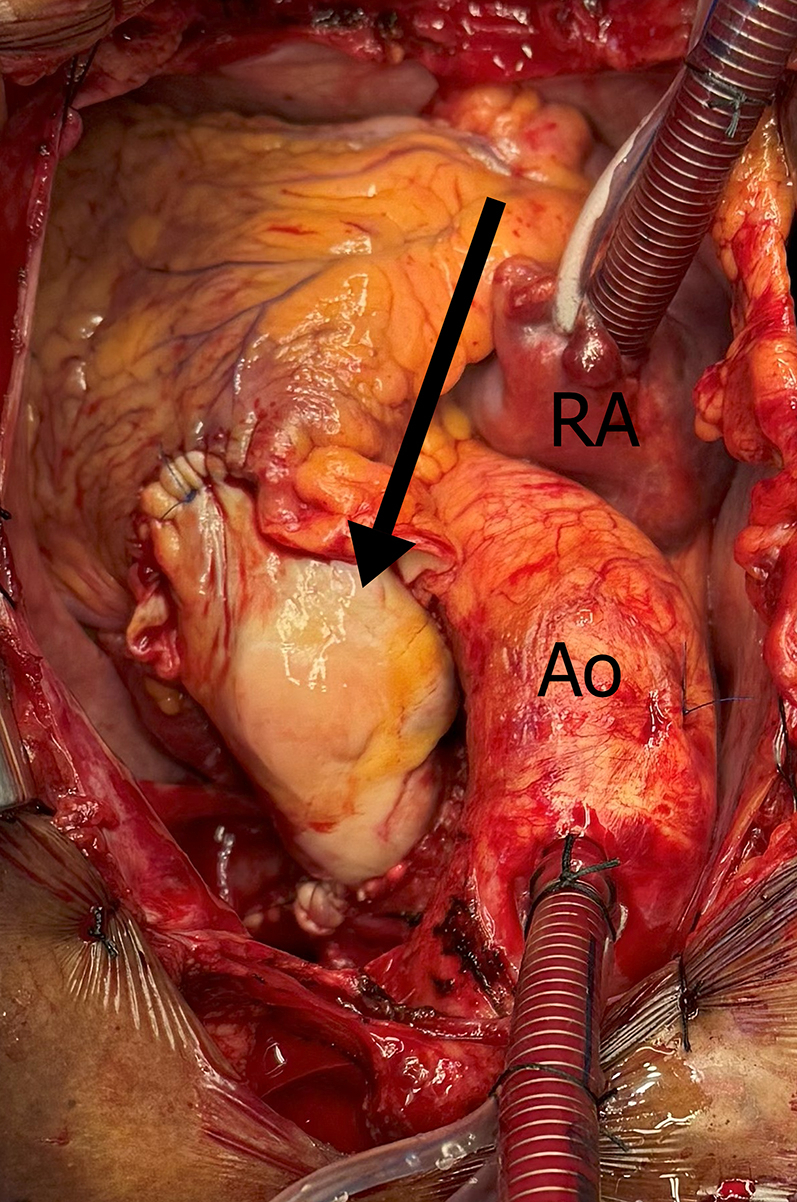
Surgical Correction of Aneurysm of the Main Pulmonary and Left Pulmonary Artery, Through Aneurysmectomy and Pulmonary Homograft Implantation (Arrow) Ao = aorta; RA = right atrium.

A multidisciplinary team decided to undergo surgical intervention. The procedure was performed through median sternotomy, with cardiopulmonary bypass time of 57 minutes and ischemia time of 35 minutes, under moderate hypothermia (30-32 °C). After resection of the aneurysm and the stenotic pulmonary valve, valve replacement was performed with a DPH (size 25), fixed with continuous polypropylene 4-0 suture, combined with plastic of the left pulmonary branch (Figure 5, Figure 6, Figure 7, Video 1).

## Anticoagulation Strategy

Intraoperative anticoagulation was achieved with intravenous heparin, targeting an activated clotting time of >480 seconds. Postoperatively, unfractionated heparin was continued until hospital discharge. No antiplatelet therapy and no long-term anticoagulation were prescribed, as DPHs do not require chronic antithrombotic therapy.

## Follow-Up

The immediate postoperative period was satisfactory, with complete symptom resolution. However, the patient developed severe pneumonia requiring prolonged ventilatory support and broad-spectrum antibiotics. During the investigation, SC hemoglobinopathy was incidentally diagnosed and confirmed by laboratory testing (Table 2). The patient was discharged after 24 days.

**Table 2:** Postoperative Laboratory Tests to Investigate Anemia

Measure	Result	Reference Value
Coombs direct	Positive	Negative
Hemoglobin A1	37.4%	>95%
Hemoglobin A2	6.0%	1.5%-3.7%
Hemoglobin fetal	1.1%	<2.0%
Hemoglobin S	30.1%	Absent
Hemoglobin C	25.4%	Absent
Others	0.0%	Absent
Iron	26.5 μg/dL	70-180 μg/dL
Ferritin	1,241.9 ng/mL	23.9-336.2 ng/mL
Total bilirubin	0.91 mg/dL	0.30-1.00 mg/dL
Direct bilirubin	0.36 mg/dL	<0.20 mg/dL
Indirect bilirubin	0.55 mg/dL	<0.80 mg/dL
Lactate dehydrogenase	1,054 U/L	<247 U/L

Follow-up chest computed tomography revealed persistent dilation of the pulmonary arteries, while echocardiography showed a competent graft, maximum gradient of 4.5 mm Hg, preserved left ventricular function, and an ejection fraction of 66% (Table 3). The patient remains under joint cardiology and hematology follow-up, without the need for anticoagulation or reintervention, and is on iron and folic acid supplementation.

**Table 3:** Findings on 2-Month Postoperative Transthoracic Echocardiogram Showing Low Gradients Through the Pulmonary Homograft

Measure	Value
Aortic root (mm)	34
Left atrium (mm)	37
Right ventricle (mm)	23
Interatrial septum (mm)	11
Posterior wall thickness (mm)	10
LV diastolic diameter (mm)	42
LV systolic diameter (mm)	27
Shortening fraction (%)	36
LVEF (Simpson%)	66
LV mass (g)/BSA	84
TAPSE (mm Hg)	—
PSAP (mm Hg)	—
Description	
Tricuspide valve	Adequate opening, minimal reflux, which prevented adequate PASP estimation.
Pulmonary valve	The allograft appeared well, with adequate mobility (PV: 1.1 m/s, mean gradient: 2.7 mm Hg, maximum: 4.5 mm Hg), and was competent.
Conclusion	The left ventricle had normal dimensions, concentric remodeling, preserved segmental contractility, and overall systolic function. The left atrium and right chambers were normal. Minimal mitral and tricuspid regurgitation was observed. The pulmonary allograft functioned normally.

BSA = body surface area; LV = left ventricle; LVEF = left ventricular ejection fraction; PSAP = pulmonary systolic artery pressure; TAPSE = tricuspid annular plane systolic excursion.

## Discussion

Idiopathic MPA aneurysm is a rare and challenging condition, especially when associated with pulmonary valve stenosis.^1^^,^^2^^,^^4^ The risk of rupture, sudden death, and compressive complications justifies surgical indication in symptomatic cases, those with rapid growth, or aneurysms >5.5 cm.^1^, ^2^, ^3^, ^4^

In this case, pulmonary valve replacement was necessary because preoperative imaging demonstrated moderate valvular stenosis with significantly elevated transvalvular gradients. Intraoperative inspection confirmed diffusely thickened cusps with restricted mobility, preventing adequate coaptation. Given these findings, valve-sparing reconstruction was unlikely to provide durable competence, and homograft implantation offered a more reliable and lasting solution.

The choice of reconstructive technique is critical in young patients. Synthetic prostheses, such as Dacron and PTFE (polytetrafluoroethylene), carry higher risks of thrombosis, endocarditis, and anticoagulation requirement, whereas conventional bioprostheses tend to degenerate faster on the right side of the heart.^4^^,^^6^

DPHs have emerged as a promising alternative, providing lower immunogenicity, reduced calcification, no need for anticoagulation, and hemodynamic performance similar to native tissue.^7^^,^^8^ Studies on human valved homografts have shown that different decellularization methods can preserve structure and reduce immunogenicity,^8^ while experimental investigations in animal models have demonstrated that different processing techniques directly affect resistance, structure, and durability.^8^^,^^9^ Recent analyses emphasize that despite these advantages, challenges remain regarding immune response, tissue integration, and postimplant complications.^10^

The choice of a DPH was particularly appropriate in this case. Beyond the patient's young age, the postoperative diagnosis of SC hemoglobinopathy added a layer of complexity. This condition is associated with pulmonary hypertension, thrombotic events, and vascular inflammation.^5^ These mechanisms could adversely affect the performance of synthetic grafts or standard bioprostheses, potentially compromising long-term outcomes.

The patient's postoperative course demonstrated satisfactory clinical and functional recovery, with a competent graft and no thromboembolic complications, reinforcing the potential benefit of this technique. Persistent arterial dilation, however, may reflect irreversible structural changes or hemodynamic repercussions of hemoglobinopathy, underscoring the need for strict follow-up. This is to our knowledge the first reported case of idiopathic MPA aneurysm in a patient with SC hemoglobinopathy treated with a DPH, contributing to the still scarce literature on this topic.^5^^,^^7^

In Brazil, DPHs are supplied through a single national multitissue bank located in our region. Although our institution actively participates in tissue procurement and processing—steps that include donor screening, serological testing, dissection, antibiotic sterilization, microbiological clearance, and cryopreservation completed within 24 to 72 hours—the intrinsic waiting period for a suitable graft remains unavoidable. The approximately 60-day interval reflects not processing time, but rather donor availability, procurement logistics, and the existing surgical queue, representing an important logistical consideration when scheduling elective procedures.

## Study Limitations

Significant scientific gaps remain, as there are no prospective randomized studies directly comparing reconstructive techniques and types of grafts, nor are there uniform criteria for surgical indication in MPA aneurysms. Centralizing treatment in referral centers with experienced teams and advanced technologies tends to optimize outcomes, reducing complications and reoperations.^4^

Despite the benefits associated with pulmonary homografts, especially in their decellularized form, which present greater durability and lower immunologic response,^8^^,^^9^ challenges persist regarding immune response, tissue integration, and postimplant complications.^10^ In addition, their use remains limited by the restricted availability of human cardiovascular tissue banks.

## Conclusions

This case demonstrates the feasibility and safety of DPHs in the surgical treatment of idiopathic MPA aneurysm, with satisfactory hemodynamic results and absence of thromboembolic complications. The association with SC hemoglobinopathy reinforces the importance of multidisciplinary management and individualized therapy in rare and complex conditions.

### Statement of Consent

Written informed consent for publication was obtained from the patient.

### Ethics Statement

This study was approved by the local ethics committee and was conducted in accordance with the Declaration of Helsinki.

## Funding Support and Author Disclosures

The authors have reported that they have no relationships relevant to the contents of this paper to disclose.

## Appendix

Video 1 Final Postoperative Appearance, With the Heart Decannulated, Showing Good Biventricular Function The white arrow shows the pulmonary homograft in the pulmonary position. Ao = aorta.
